# Supplementary material for: The causal role of circulating inflammatory markers in osteoporosis: a bidirectional Mendelian randomized study
Source: Front Immunol. 2024 Jul 18;15:1412298. doi: 10.3389/fimmu.2024.1412298 (PMC11291241; doi:10.3389/fimmu.2024.1412298)
Supplement: Supplementary file 1 [file DataSheet_1.zip › supplementary material/OSTEOPOROSIS-CIM/GCST90274809.tsv.gz_leave_one_out.pdf]

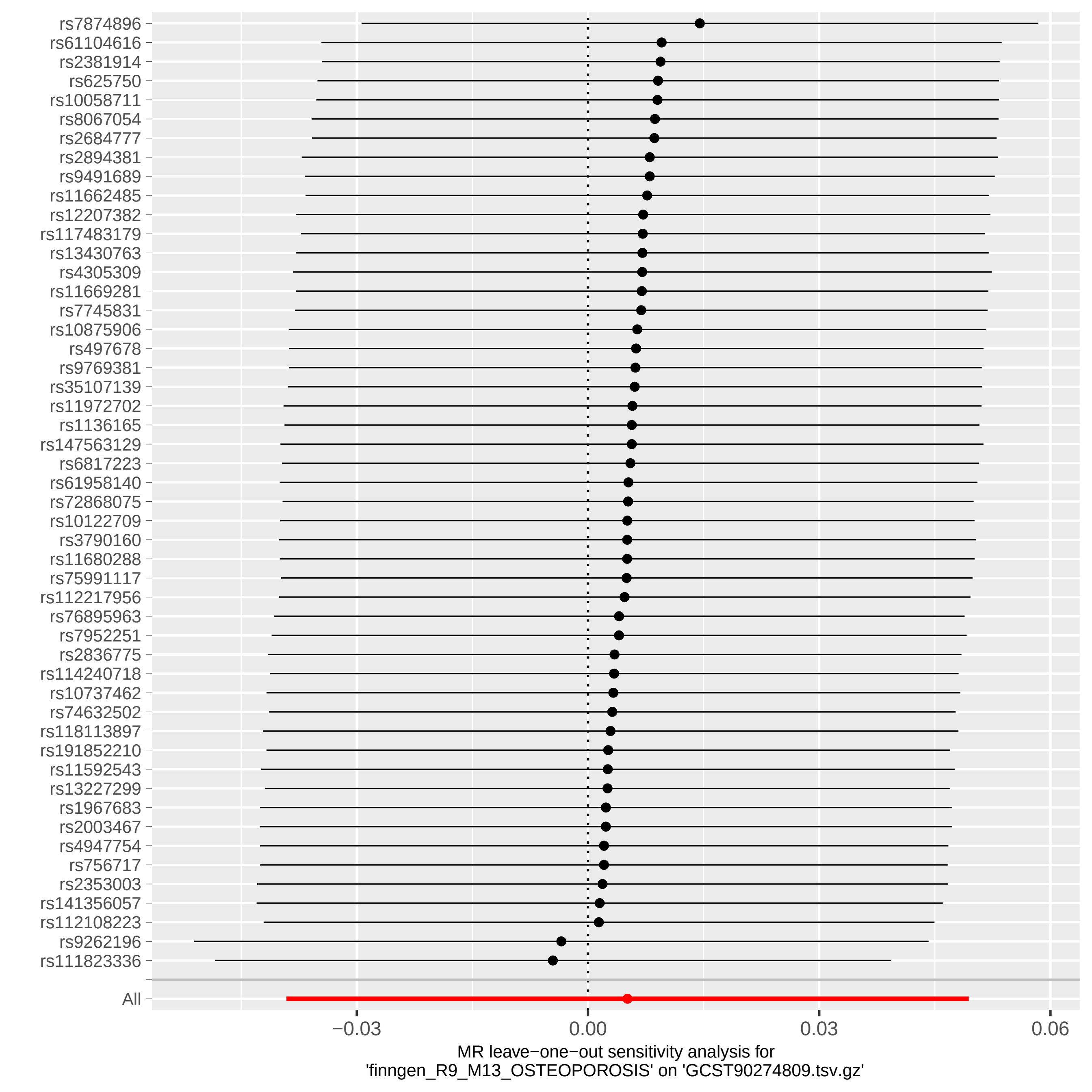
rs7874896
rs61104616
rs2381914
rs625750
rs10058711
rs8067054
rs2684777
rs2894381
rs9491689
rs11662485
rs12207382
rs117483179
rs13430763
rs4305309
rs11669281
rs7745831
rs10875906
rs497678
rs9769381
rs35107139
rs11972702
rs1136165
rs147563129
rs6817223
rs61958140
rs72868075
rs10122709
rs3790160
rs11680288
rs75991117
rs112217956
rs76895963
rs7952251
rs2836775
rs114240718
rs10737462
rs74632502
rs118113897
rs191852210
rs11592543
rs13227299
rs1967683
rs2003467
rs4947754
rs756717
rs2353003
rs141356057
rs112108223
rs9262196
rs111823336
All
−0.03
0.00
0.03
0.06
MR leave−one−out sensitivity analysis for
'finngen_R9_M13_OSTEOPOROSIS' on 'GCST90274809.tsv.gz'
